# Medical Opinion and Movement

**Published:** 1908-12-05

**Authors:** 


### Medical Opinion and Movement.

INJECTIONS of gelatine solution were first introduced by Lancereaux in the treatment of aneurysm, and although several successful results were reported, a more extended application of the treatment did not increase the confidence of the profession in its efficacy. It was, however, generally recognised that the injections increased the coagulability of the blood, and their consequent application to cases of severe hæmorrhage has met with more general approval. M. Chaput has recently reported on the results he has obtained in cases of operative hæmorrhage, and he is of opinion that gelatine injections are especially indicated in cases of secondary hæmorrhage. He cited several cases of hysterectomy and removal of large uterine fibromata, in which troublesome hæmorrhage yielded speedily to gelatine injections. When secondary hæmorrhage sets in, instead of immediately opening the wound, M. Chaput advises the injection of about 5 grammes of gelatine in a 1 per cent. solution first, and then, when the hæmorrhage has in consequence ceased, the search can be made for the cause with greater chance of success.

---

THE treatment of gastro-intestinal disorders by means of the lactic acid producing bacilli is already well known, and may be said to be now on its trial by the profession in general. The pharmacist has also met the requirements of the profession by various preparations containing the bacilli in concentrated form, the most convenient of which is, perhaps, the compressed tablet. Dr. W. H. Walsh, of Philadelphia, claims that a new field of usefulness has been found for these preparations, and advocates their use in concentrated solutions for the treatment of chronic catarrh and other affections of the ear, throat and nasal passages and sinuses. A liquid containing these bacilli sprayed into these cavities is said to destroy the pathogenic and saprophytic bacteria, with which it comes in contact, and quickly to relieve the morbid condition. It is claimed that the lacto-bacilli proliferate within the cavities, and finding a culture medium in the secretions penetrate where it is almost impossible to apply other local remedies. Dr. Walsh advises that the clear fluid from Bulgarian butter-milk be used as the culture medium for injection or spraying. The following are his directions: Take 500 c.c. of pure milk, remove the cream, add 10 grammes of cane sugar and boil for a quarter of an hour, cool to 42° C., and add one tablet of the lacto-bacilli, crushed. Keep for ten hours in a water bath at 42° C., when the butter-milk will be formed thick and creamy. Place in a refrigerator until a fluid separates, and syphon off the clear fluid from the bottom. This fluid is rich in bacilli, sugar, and peptones, and is ready for use in the manner suggested. Even though further trials prove its efficacy, the trouble involved in its preparation will be a serious deterrent to its use in private practice, although there should be no difficulty at institutions with a bacteriological laboratory.

AT a meeting of the Liverpool Medical Institution the value of fibrolysin was discussed, and its power of causing absorption of cicatricial tissue. Dr. Karl A. Grossmann appears to have obtained very successful results with its use, especially in ophthalmic practice, and reported on several cases in which the administration of the drug was followed by marked improvement in the condition. He cited a case of severe burning of the eyelids, in which a thick dense scar had formed and prevented movement of the eyelid, causing some ulceration of the cornea. After a course of treatment the scar tissue was so far absorbed that the lid resumed its normal power of opening and closing, and there remained only a small amount of shortening. Dr. R. J. M. Buchanan also gave his experience of the drug in a case of chronic sclerosis of the spinal cord of several years' standing. After the administration of the drug the contractures and pain were much diminished, there was less ataxia, and the power of walking returned. He stated further that he had found injections of fibrolysin of value in cases of non-tuberculous pleural adhesions. The injections must be made deeply under the skin, otherwise the drug is apt to produce punched-out sores.

---

THE much-vexed question of the pathology of gastric ulcer has not yet emerged from the sphere of speculation and hypothesis. Last week reference was made to the experimental work of Katzenstein, which seemed to suggest that normally the cells of the gastric mucosa are possessed of an "antipepsin" capable of preventing their own digestion by the gastric juice, and that gastric ulcer was caused by a deficiency of this substance in the affected cells. Dr. Charles Bolton's observations, however, have led him to a contrary conclusion. In a paper entitled "The Mode of Action of Gastrotoxic Serum and the Healing of Gastrotoxic Ulcers," read before the Royal Society of Medicine, he gives the results of his researches. He prepared a gastrotoxic serum by immunising the rabbit with the guinea-pig's gastric cells. The serum injected produced general symptoms of intoxication, and also patches of necrosis in the gastric mucous membrane which developed into ulcers. Neutralisation of the gastric juice with alkali prevented necrosis taking place. From this Dr. Bolton concludes that the serum devitalises the cells and so renders them a prey to the action of the gastric juice. He does not consider that the change produced in the cells was due to the removal of any specific resisting power of the cells, because other poisons—hepatotoxin, enterotoxin, and hæmolysin have the same effect, though to a less extent. It may be doubted, however, whether this fact altogether excludes the possibility of the gastric cells possessing some anti-body which normally immunises them against the action of the gastric juice, as suggested by Katzenstein. Further research on these lines may elucidate the problem and lead to some therapeutic means of dealing with the disease of gastric ulcer.

THE New York Post-graduate Medical School benefits under the will of the late Mr. Frederic Cooper Hewitt, of New York, to the extent of two million dollars. A class-mate of the late Dr. Roosa, whose name is indelibly associated with the advancement of the post-graduate movement in America in general and the New York Post-graduate School in particular, Mr. Hewitt recognised early the splendid work which the school is doing and was always "a friend of his friends' friends." "He realised," remarks our contemporary, the official organ of the school, "what so few seem to have done, that the great need was not new institutions, but the strengthening financially of those which already had proved their worth, thus placing in the hands of the managers of these, the means of further development and expansion. He was large-minded enough not to be influenced by the ambition to have his name carved over some doorway. . . . And this fact which stands forth is that no restrictions were attached to his bequests. He showed his faith in the institutions which he deemed worthy of his benevolence by decreeing that his estates should be sold and his legatees paid in cash." The post-graduate study movement has warm friends across the Atlantic, and we can only hope that their example may stimulate others here to follow in their steps and support graduate study in England.

---

WE have already on more than one occasion referred in these columns to the Bier treatment of inflammatory conditions by the inducement of passive hyperæmia. While the method is not the panacea for all ills which some of Professor Bier's pupils make it out to be, there is no doubt that it is a most useful method, which, owing to its ease of application, its inexpensiveness and its catholicity, is specially worthy the attention of the general practitioner. One of the most recent tributes to its usefulness is a paper which Frank contributes to the *Medizinische Klinik*, recording his experience of the method in cases of gonorrhœa and other urogenital conditions. His paper is the more valuable because he had tried the treatment some years ago and discarded it, as he had seen no good results from it. This want of success, he admits, was doubtless due to a faulty technique in employing the treatment; certainly his later experiences are in every way encouraging. In gonorrhœal rheumatism, for instance, the application of the bandage acts promptly, relieving the patient from pain, while in cases of tubercular disease of the genito-urinary tract the success obtained is in many instances striking. In cases of tuberculous disease of the testicle he employs a modified suction-cup which fits over the scrotum. Bier himself recommends the application of an elastic bandage encircling the cord and base of the scrotum and put on moderately tightly. Either method will serve, and when the treatment is properly carried out it is the most simple and one of the most effective measures which the practitioner has at hand in dealing with chronic or acute inflammatory scrotal conditions.

JEAUNIN reports several cases of puerperal infection successfully treated by injections of colloidal silver. A point upon which he lays stress is that all the cases treated were bad cases. The paper is full of information, and frankly relates the failures as well as the successes. In all 43 cases were treated, with a mortality of 24 per cent., a figure which is very satisfactory when it is remembered that all the cases were cases of general puerperal septicæmia in which the prognosis was very serious. Jeaunin claims that colloidal silver, or collargol, as it is more generally known, is a powerful germicide, antiseptic, and catalytic ferment, which is, moreover, non-poisonous. He uses it in the form of injections, and not, as first advocated, as inunctions. Usually 10 cm. of a 1 per cent. solution is injected intravenously, ordinary precautions being observed in making the injection. The result of the injection is usually a marked reaction; the patient has a rigor and her temperature rapidly rises, only, however, to fall within a few hours. If the temperature remains high the prognosis is unfavourable. A second injection may be given in that case, and even a third, but in such cases the drug appears to have little effect. The author writes enthusiastically about the treatment, and recommends it as a routine line in every case where there is danger of the infection becoming generalised. Other observers have reported favourably on the use of collargol in the treatment of septic conditions, but at present the method appears to be confined to French clinics.

---

THE researches of various investigators into the subject of placental syphilis and its connection with the spirochœta pallida have resulted in indecisive and contradictory findings; so Dr. W. O. Pauli has undertaken independent work, the conclusions from which he publishes in the *Johns Hopkins Hospital Bulletin*. Some twenty-five placentæ were diagnosed microscopically as syphilitic by Professor J. W. Williams, and autopsies on sixteen of the fœtuses showed naked eye evidence of syphilis in fourteen of them. The microscopical characters of syphilitic placentæ are, less delicate branching than normally of the villi; the ends of the finer branches thickened and club-shaped; blood vessels absent in the finer branches and poorly seen in the main villous stems; and an opaque and granular appearance of the stroma. In not a single placenta was the spirochœta found, despite the most careful search, though it was detected in large numbers in the internal organs of 11 out of the 16 bodies upon which autopsy was permitted. The inferences drawn are that the placental changes are the results of toxins produced by the micro-organism in the fœtal organs, and that the placenta is not the nidus of infection. Incidentally it may be noted that the series of cases illustrates very well the ratio as to mass that exists between a syphilitic fœtus and its placenta. This ratio is normally about six to one, but in syphilis is generally four to one, or even less. Among the 25 cases examined there were but two wherein this ratio was as much as five to one, and one of these was the only one without gross anatomical syphilitic lesions.

TESTS for bile constituents in urine are usually not very satisfactory in private practice when all the various reagents are not at hand. The nitric acid test is one of the most unsatisfactory of all, and it is, besides, a particularly unpleasant one when performed in a small surgery. Yet it is often very useful to detect the presence of small quantities of bile constituents in urine. A positive result usually helps towards the diagnosis: a negative result, though not so useful, is sometimes helpful. Popper and Obermayes have recently taken up the subject of tests for bile pigments, and have stated their results in an interesting article in the *Wiener Klinische Wochenschrift*. They recommend the employment of a test solution consisting of water 625 c.c., alcohol (95 per cent.) 1.25 c.c., sodium chloride 1,100 grams, potassium iodide 180 grams, and 10 per cent. solution of tincture of iodine 3.5 c.c. Five cubic centimetres of this test solution is put into a test-tube and the urine to be examined is carefully poured on to the surface. If bile is present a blue-green ring forms at the zone of contact: if the urine contains merely a trace of bile the ring is faintly blue in colour. The test solution keeps for an indefinite time, is always ready for use, and appears to be excellent for ordinary purposes.

---

THE experiments of M. F. Erben (*Prager Med. Woch.*) in the analysis of the blood of diabetics, show that the quantity of lecithin is diminished in the red corpuscles, but remains normal in the plasma. Diabetics are thus unable to utilise phosphorised fat in the formation of their erythrocytes. The author believes that there is a real defect in the constitution of the blood elements and inclines to think that this defect extends to all the cells of the organism. If his opinions are correct, the favourable effect of lecithin in the treatment of diabetes is explained. Lancereaux was the first to introduce this treatment, and his experience is that under the influence of the drug the patients increase in body weight and improve in general health. M. Erben thinks, however, that the doses recommended by Lancereaux are not sufficiently large. He prescribes .4 to .5 gram daily and administers the drug subcutaneously.

---

IT is generally held that retention of the membranes is the source of many accidents after childbirth, and necessitates active intervention; but an article in the *Centr.-Bl. f. Gynäkol*, by Dr. E. Engelhorn, may somewhat modify our views in this respect. During the last seven years, at the clinic of Erlangen, the author has collected 64 cases of retained membranes out of 2,377 accouchements, or 2.7 per cent. Of these 64 cases, 11 were the result of uterine expression. In 34 cases a rise of temperature was noted, which only lasted for 24 hours in 26 of the cases, and in the remaining 8 cases only lasted a few days. All the women were able to leave hospital on the twelfth day. Treatment, where adopted, consisted in the administration of ergot and vaginal douching; but in most cases none was necessary. In only two cases was active intervention adopted, one woman being relieved of the membranes digitally, the other receiving an intra-uterine douche. For the most part treatment in no way accelerated the convalescence of the patients. The author maintains that douches are not, on the whole, to be recommended after childbirth. He believes that retention of the membranes is an accident of which the dangers have been exaggerated, and that in most cases it is best to adopt no active intervention at all. Risk of infection of the patient is augmented by digital or instrumental interference.

---

ACCORDING to Etterlen in the *Lyon Medical*, alkaline urine tends to break down the urinary mucus, and this decomposition in turn tends to render the urine more alkaline. He insists that alkaline urine, whether infected or not, should always be acidified. In phosphaturia acidification is necessary since phosphates are not precipitated in acid urine. To effect this the author has found boric acid the most useful drug. He gives it in doses of from 1 to 2 grams daily, either in cachets or in solution. When given for several days in 2-gram doses, a considerable reduction of pus or of phosphates is noticeable. He maintains that boric acid is more useful than benzoic or hydrochloric acid as an acidifier. It is moreover, a diuretic and thus favours a beneficial flushing of the kidney and the whole organism. The alleged toxicity of boric acid need not prejudice this line of treatment, since the author has only been able to discover five fatal cases of poisoning in the whole of medical literature.

---

DAWBARN draws attention to a means of diagnosing the presence of a piece of muscle between the fragments of a fracture. Absence of crepitus cannot be meant to prove such a complication, and the presence of grating is no sure proof that the fragments are in apposition without any intermediate soft tissues. It is obviously of great importance to diagnose the condition, since the success of treatment to a large extent depends upon correct apposition. Hitherto surgeons have largely guessed the complication by trusting to the absence or presence of definite crepitus, but the author points out that the stethoscope may give useful information. "In the case of a fracture of the shaft of the humerus, the existing condition can be determined in a few minutes by placing a stethoscope over the greater tuberosity of the bone while an assistant percusses over the external condyle. When there is fairly good apposition of the fragments and no torn tissues have slipped between the ends, upon comparing the sounds by this method, first on the normal and then on the broken bone, very little difference is noticeable. In a case where no union or very poor union must result (owing to interposition of soft tissues) no surgeon with fairly good hearing can fail to recognise a striking discrepancy in both intensity and pitch upon the two sides. This method, which the author calls "Osteophonic percussion," is decidedly novel, and ought to be of value in fractures of the long bones.

